# Time-Course Transcriptome Analysis of Gingiva-Derived Mesenchymal Stem Cells Reveals That *Fusobacterium nucleatum* Triggers Oncogene Expression in the Process of Cell Differentiation

**DOI:** 10.3389/fcell.2019.00359

**Published:** 2020-01-14

**Authors:** Wenyan Kang, Tianyong Sun, Di Tang, Jiannan Zhou, Qiang Feng

**Affiliations:** ^1^Department of Human Microbiome, School and Hospital of Stomatology, Shandong University, Shandong Provincial Key Laboratory of Oral Tissue Regeneration, Shandong Engineering Laboratory for Dental Materials and Oral Tissue Regeneration, Jinan, China; ^2^Department of Periodontology, School of Stomatology, Shandong University, Jinan, China; ^3^State Key Laboratory of Microbial Technology, Shandong University, Qingdao, China

**Keywords:** *F. nucleatum*, GMSCs, time-series RNA-seq, TAGs, transcription factor, microRNA

## Abstract

*Fusobacterium nucleatum* has pathogenic effects on oral squamous cell carcinoma and colon cancer, while the effects of continuously altered gene expression in normal human cells, as induced by persistent exposure to *F. nucleatum*, remain unclear. In this study, a microarray Significant Profiles (maSigPro) analysis was used to obtain the transcriptome profile of gingiva-derived mesenchymal stem cells (GMSCs) stimulated by *F. nucleatum* for 3, 7, 14, and 21 day, and the results revealed 790 (nine clusters) differentially expressed genes (DEGs), which were significantly enriched in cell adherens junctions and cancer-related pathways. On the basis of a short time-series expression miner (STEM) analysis, all the expressed genes in the GMSCs were grouped into 50 clusters according to dynamic gene expression patterns, and the expression levels of three gene clusters in the *F. nucleatum*-treated GMSCs were significantly different than the predicted values. Among the 790 DEGs, 50 tumor-associated genes (TAGs; such as L3MBTL4, CD163, CCCND2, CADM1, BCL7A, and IGF1) and five core dynamic DEGs (PLCG2, CHI3L2, L3MBTL4, SH2D2A, and NLRP3) were identified during *F. nucleatum* stimulation. Results from a GeneMANIA database analysis showed that PLCG2, CHI3L2, SH2D2A, and NLRP3 and 20 other proteins formed a complex network of which 12 genes were enriched in cancer-related pathways. Based on the five core dynamic DEGs, the related microRNAs (miRNAs) and transcription factors (TFs) were obtained from public resources, and an integrated network composed of the related TFs, miRNAs, and mRNAs was constructed. The results indicated that these genes were regulated by several miRNAs, such as miR-372-3p, miR-603, and miR-495-3p, and several TFs, including CREB3, GATA2, and SOX4. Our study suggests that long-term stimulation by *F. nucleatum* may trigger the expression of cancer-related genes in normal gingiva-derived stem cells.

## Introduction

The oral cavity is the home of microbial communities that live in symbiosis with one another and play essential roles in maintaining a normal oral physiological environment in healthy individuals (Duran-Pinedo et al., 2014). The imbalance of the oral microbiome is related to many diseases in addition to periodontal disease and dental caries, such as a series of cancer and infectious diseases induced directly by chemical carcinogen or virulent protein metabolisms (Abiko et al., 2010; Meurman, 2010). Chronic periodontal disease initiated by the dysbiosis of plaque microorganisms has been recognized as a crucial risk factor for oral precancerous lesions and cancers (Tezal et al., 2009; Gondivkar et al., 2013). Periodontitis-related pathogens are also closely linked with various cancers, such as esophageal, gastric, prostate, and colorectal cancer (Fitzpatrick and Katz, 2010; Yao et al., 2014). Previous studies have suggested that gingival squamous cell carcinoma is clinically similar to various inflammatory diseases, such as periodontitis, periodontal/endodontic lesions, and dentoalveolar abscess (Kirkham et al., 1985; Heller et al., 1991; Levi et al., 2005; Yoon et al., 2007).

As a high-frequency periodontal pathogen, *Fusobacterium nucleatum* (*F. nucleatum*) is closely related to the occurrence and development of periodontitis, and a recent study suggested that it plays key roles in the carcinogenesis of colon cancer (Socransky et al., 1998; Gholizadeh et al., 2017). *F. nucleatum* can produce a group of virulence factors and toxic metabolites, such as lipopolysaccharides, porins, butyrate, and propionate ammonia, which are beneficial for invasive tissues and contribute to pathogenicity (Bolstad et al., 1996; Jeng et al., 1999; Narayanan et al., 2002). *F. nucleatum* is closely related to colorectal cancer, pancreatic cancer, and oral cancers (McCoy et al., 2013; Mitsuhashi et al., 2015; Gholizadeh et al., 2016). *F. nucleatum* can promote the initiation and development of colorectal cancer by adhering to cells through its unique FadA protein, which can bind to endothelial cells through a special vascular endothelial cadherin receptor (CDH5) and interact with epithelial cells through E-cadherin, which then activates subsequent β-catenin signaling and increases the expression levels of inflammatory genes, transcriptional factors, and oncogenes (Fardini et al., 2011; Rubinstein et al., 2013). In oral squamous cell carcinoma (OSCC) tissues, *F. nucleatum* levels were significantly elevated and mainly detected at deep and overlaying portions of the tumor (Hooper et al., 2006, 2007; Pushalkar et al., 2012). Moreover, the Fap2 protein of *F. nucleatum* can directly interact with the human inhibitory receptor TIGIT, which is present on all human natural killer (NK) cells and T cells, leading to the inhibition of NK cell killing of tumor cells (Gur et al., 2015).

Novel and promising tissue engineering strategies based on stem cells have shown great potential to rescue periodontitis tissue destruction (Hu et al., 2018). Gingiva-derived mesenchymal stem cells (GMSCs) have been confirmed as a promising cell source and can play immunomodulatory and anti-inflammatory functions *in vivo* (Zhang et al., 2009). GMSCs have been successfully used in repairing minipig severe maxillofacial bone defects and RGD-modified alginate hydrogels loaded with GMSCs have had excellent effects in bone tissue engineering applications (Diniz et al., 2016; Shi et al., 2019). During the process of periodontal tissue regeneration, it is difficult to absolutely remove pathogenic microorganisms, and recent studies have been flawed because the existing inflammatory environment in the destroyed areas was not considered. Therefore, effect of the inflammatory environment on dental tissue repair cells remains unknown. In our current study, we constructed an *in vitro* experimental model of long-term *F. nucleatum* stimulation of GMSCs during the process of osteogenic differentiation. This study was designed to answer questions regarding changes in gene expression in tissue repair cells that are continuously stimulated by oral pathogens.

High-throughput RNA sequencing (RNA-Seq) has been widely used to analyze transcriptomic changes across the whole genome (Ozsolak and Milos, 2011; Raz et al., 2011). Due to the outstanding advantages of sensitive, efficient, and accurate detection of digital method of all expressed genes, RNA-Seq has been widely used to identify the potential transcriptional mechanisms and components of oral cancers; for example, it has been used to analyze the initiation of cancer stem cells in patients with head and neck squamous cell carcinoma, and for identifying specific global gene expression signatures of tongue squamous cell carcinoma (Salazar-Garcia et al., 2018; Zhang et al., 2018). In the present study, we used time-course RNA-Seq transcriptomic analysis to identify the tumor-associated gene (TAG) and core dynamic differentially expressed gene (DEG) signatures affected by time-series *F. nucleatum* stimulation. Additionally, we constructed an integrated network containing dynamic DEGs, transcriptoin factors (TFs), and microRNAs (miRNAs). Our study revealed the alteration of gene expression across the genome in time-series *F. nucleatum* stimulation of GMSCs, which lays a foundation for understanding the cellular changes induced by long-term stimulation of oral pathogens.

## Materials and Methods

### Microarray Data

The mRNA expression profile of GSE126821 in the GEO database^1^ has been reanalyzed, which has been deposited according to our previous study (Kang et al., 2019). Briefly, three healthy individuals who underwent impacted tooth extraction at Stomatology Hospital of Shandong Province were recruited to participate in this study. The GMSCs from the three donors were successfully isolated, cultured in osteogenic medium, and stimulated by *F. nucleatum* (MOI = 100) at 3, 7, 14, and 21 day. The non-stimulated GMSCs were served as negative control at each time point. Finally, a total of 24 samples were collected and sequenced to analyze the expression level across the genome at Novogene (Beijing, China) by RNA-seq.

### Preprocessing of the Raw Data

The raw count expression data (19847^∗^24 matrix) were transformed into logarithm values of log_2_(x+1) using R software (version 3.6.1). The protein-coding genes were extracted, and genes with low expression levels (gene expression value equal to zero in more than 20 samples) were removed from the gene expression set. Finally, we achieved a comprehensive profile comprising 16705 genes with 24 slides (16705^∗^24 matrix).

### Time-Course Differential Expression Gene (DEG) Analysis

Time-course DEG analysis was performed according to the R package microarray Significant Profiles (maSigPro) (version 1.56.0) (Conesa et al., 2006). Briefly, to obtain the candidate genes, the least squares method was used to determine the coefficients of each independent variable, and the significance of the equation was evaluated according to the *F*-test. Then, stepwise regression was used to determine the best combination of independent variables and screen the significant genes, which led the comprehensive time-course DEGs.

### Gene Ontology (GO) and Kyoto Encyclopedia of Genes and Genomes (KEGG) Pathway Enrichment Analyses

With data on the time-course DEGs in the *F. nucleatum*-treated samples and the control samples, we used maSigPro, R package clusterProfiler (version 3.10.1) (Yu et al., 2012), to identify the overrepresented GO categories and the significant KEGG pathways. Significant pathway enrichment in the GO and KEGG databases was determined according to the selection threshold value *q* < 0.05 and gene counts ≥2. Significant KEGG signaling pathways were visualized by the R Pathview package (version 1.22.3) (Luo and Brouwer, 2013).

### Screening of the Tumor-Associated Genes (TAGs)

The entire group of TAGs was obtained from the Comparative Toxicogenomics Database (CTD)^2^ according to the key words “cancer” and “tumor”. A Venn analysis^3^ was used to identify the TAGs that overlapped among the DEGs and TAGs candidates. The expression levels of the overlapped genes were presented by a heatmap, and the most significant DEGs were further validated by a real-time polymerase chain reaction (qRT-PCR) experiments. Moreover, the gene expression levels of CCND2 and CD163 in OSCC cells were also evaluated according to the Oncomine database^4^ (Rhodes et al., 2004).

### Short Time-Series Expression Miner (STEM) and Functional Similarity Analyses

Short time-series expression miner software (version 1.3.8) (Ernst and Bar-Joseph, 2006) was used to identify the dynamic gene expression clusters in GMSCs with or without *F. nucleatum* infection, and the statistically enriched gene families with similar expression patterns were assessed according to the default parameter. A functional enrichment analysis of the statistically significant clusters was performed using the Metascape database^5^ (Zhou et al., 2019). The similar significant gene clusters in the control or the *F. nucleatum-*stimulated GMSCs were selected according to the STEM analysis results. The functional similarity between the different significant clusters was analyzed by the R GOSemSim package (version 2.8.0) (Yu et al., 2010) using Wang and Resnik scores.

### Screening of Dynamic DEGs and Constructing the Functional Network

To identify *F. nucleatum*-infected DEGs in a time dependent manner, we performed an overlap analysis of the DEGs (obtained by maSigPro) and the genes in the significant clusters of the *F. nucleatum*-infected GMSCs (obtained by STEM). Based on the overlap analysis results, the functional network was constructed according to the GeneMANIA database^6^ (Montojo et al., 2014). The functions of the genes in the functional network were determined using the Metascape database (Zhou et al., 2019).

### Transcription Factor (TF) and MicroRNA-Regulated Network Construction

The miRNAs regulating the dynamic DEGs were predicted by the miRWalk 2.0 database^7^ (Dweep et al., 2014) according to the miRNA databases-related and miRNA prediction methods, such as miRanda, PicTar, PITA, TargetScan, miRWalk, MicroT4, mirBridge, miRDB, miRMap, miRNAMap, RNA22, and RNAhybrid. The results acquired through more than six databases or the methods described above were recognized as significant miRNA-target pairs. The network was visualized by Cytoscape software (version 3.7.2) (Kohl et al., 2011). Furthermore, TF-target pairs were predicted based on the iRegulon plug-in (Version 1.3)^8^ (Janky et al., 2014) in Cytoscape according to the several parameters: minimum identity between orthologous genes of 0.05 and maximum false discovery rate on motif similarity of 0.001. Results with NES scores >5 were recognized as significant TF-target pairs and added to the miRNA-target network.

### Quantitative Real-Time PCR (qRT-PCR)

Total RNA was extracted with Trizol^®^ (CWBIO, Being, China), and mRNA concentration was determined using an ultra-micro spectrophotometer (Thermo, Waltham, MA, United States). mRNA (1 μg) from each sample was reverse transcribed to obtain the cDNA, and qRT-PCR was performed using UltraSYBR Mixture (CWBIO) on a LightCycler 96 Real-Time PCR system (Roche, Basel, Switzerland). Every sample was performed in triplicate. Data was processed according to the 2^(–ΔΔCt)^ method. The primers used for amplification in this study are shown in Supplementary Table S1.

## Results

### Cluster Analysis

**FIGURE 1:**
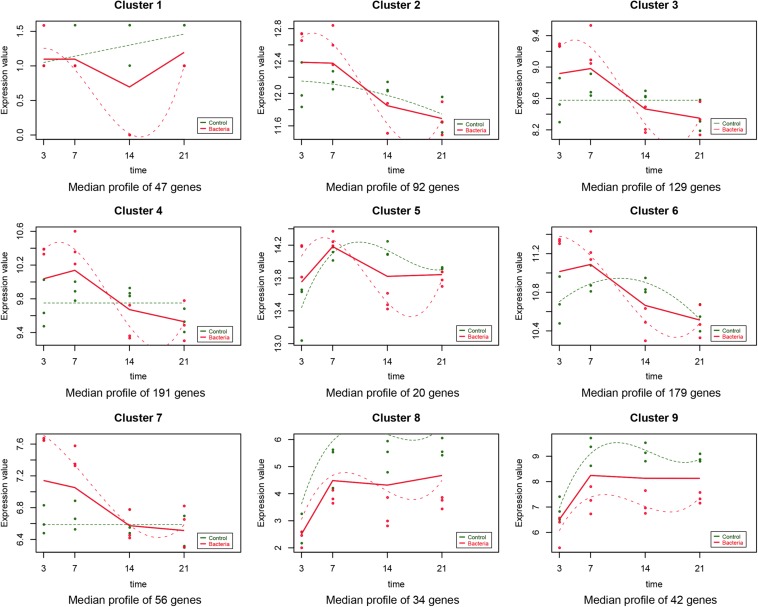
Data visualization according to the cluster analysis. Each plot shows the average expression profile of the gene clusters from all samples. Dots show the actual average expression values for each sample (green and red dots represent the control and *F. nucleatum* groups, respectively). Fitted curves of the control and *F. nucleatum* groups are displayed as green and red dotted lines, respectively. The solid line (red) has been drawn to show the actual average value of the gene expression at each time point for *F. nucleatum*-stimulated group.

As described in the section “Materials and Methods,” the experiment-wide gene expression profiles, including 24 samples from three different individuals for which the gene expression levels across the whole genome for the 24 samples were obtained by RNA-Seq. In addition to this, 790 significant time-course DEGs were identified and divided into nine clusters (Supplementary Table S2). The homogeneity of the obtained clusters is shown in Supplementary Figure S1. The mean profile for each group in each cluster and the actual profile differences between the experimental and control groups are presented in Figure 1. Compared to the untreated group, the gene expression in each cluster of the *F. nucleatum*-stimulated group varied within a certain pattern: in cluster 1, the gene expression level of the *F. nucleatum*-stimulated group was equal to that of the untreated group from 3 to 7 day, while it was downregulated from 7 to 21 day; in clusters 2, 3, and 4, the expression tended to increase at 3 and 14 day, while it decreased from 14 to 21 day; in clusters 5 and 6, the gene expression was upregulated from 3 to 7 day, and then decreased from 7 to 21 day; in cluster 7, the gene expression was upregulated from 3 to 14 day, and then tended to be stable from 14 to 21 day; and in clusters 8 and 9, the gene expression was downregulated throughout the duration of the analysis (Figure 1).

### GO, KEGG and Pathview Enrichment Analyses

**FIGURE 2:**
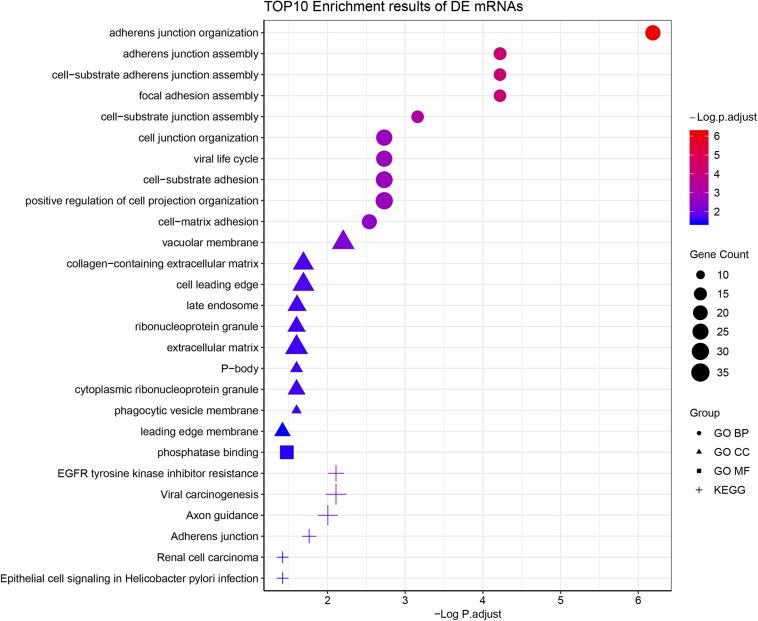
Functional enrichment analysis of all time-course DEGs. GO terms (including biological process, cellular component, and molecular function) and KEGG pathway analysis of the 790 significantly time-course DEGs. The different symbols represent the different enrichment patterns based on the GO and KEGG analyses. The size of the symbol represents the gene counts enriched in the signaling pathway. The color indicates the degree of significance.

**FIGURE 3:**
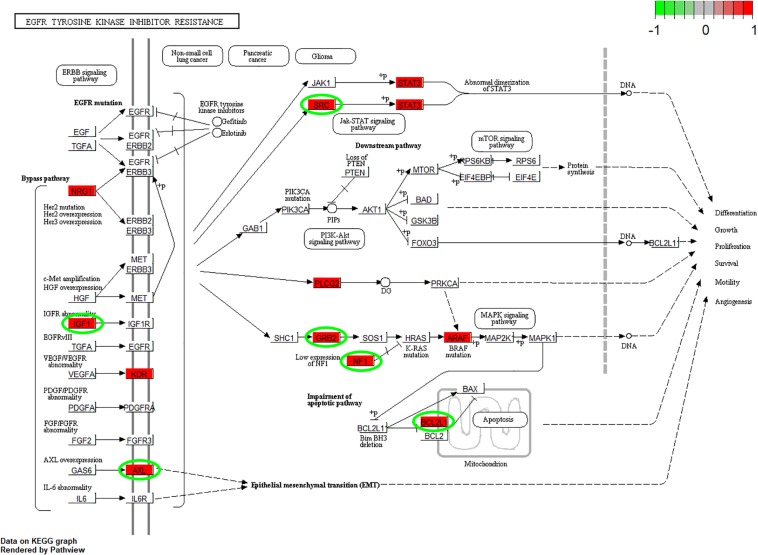
Pathview analysis of the EGFR tyrosine kinase inhibitor resistance signaling pathway. The genes in the red rectangle are time-series DEGs after cells are subjected to *F. nucleatum* stimulation. The genes with green circles are carcinogenesis related DEGs.

According to the GO enrichment analysis, the genes in the 12 clusters were annotated mainly to 67 biological process GO terms, 16 cellular component GO terms, and 1 molecular function GO term (Supplementary Table S3). In addition, all these genes were enriched in the six signaling pathways according to the KEGG enrichment analysis. The top ten GO terms and enriched KEGG pathways are shown in Figure 2. The results indicated that cell adhesion-related pathways, such as those related to adherens junction organization and adherens junction assembly, and carcinogenesis-related pathways, including EGFR tyrosine kinase inhibitor resistance and viral carcinogenesis, were related to the predominant biological processes and KEGG pathways. The results from the Pathview analysis showed that 11 genes were activated in EGFR tyrosine kinase inhibitor resistance pathways, and six of these genes were cancer-related genes (Figure 3). The significant genes enriched in axon guidance, adherens junction, epithelial cell signaling in helicobacter pylori infection, viral carcinogenesis, and renal cell carcinoma signaling pathways are shown in Supplementary Figures S2–S6.

### Screening of the TAGs

**FIGURE 4:**
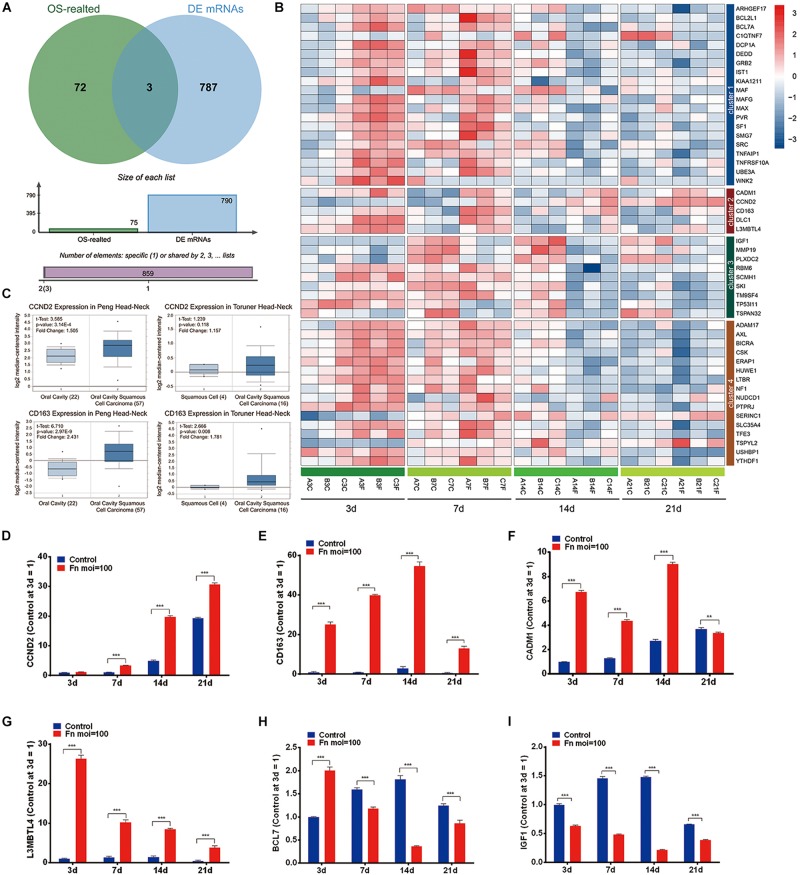
Screening of the TAGs. **(A)** Venn analysis of the carcinogenesis genes for all the TAGs and all significant time-series DEGs (cancer-related genes: all TAGs in the CTD database; DEGs: all co-regulated DEGs). **(B)** Heatmap of the 50 overlapping DEGs. **(C)** The expression levels of the CCND2 and CD163 genes in the normal oral cavity and the oral squamous cell carcinoma. The levels of gene expression of CCND2 **(D)**, CD163 **(E)**, CADM1 **(F)**, L3MBTL4 **(G)**, BCL7 **(H)**, and IGF1 **(I)** with *F. nucleatum* stimulation (MOI values of 0 and 100) at 3, 7, 14, and 21 day, as determined by qRT-PCR. ^∗∗^*P* < 0.01 and ^∗∗∗^*P* < 0.001.

The overlap analysis of all TAGs in the CTD database and the 790 time-course DEGs showed that 50 TAGs, including CADM1, CCND2, CD163, DLC1, and L3MBTL4 were identified (Figures 4A,B). Compared with the gene expression in the normal GMSCs at each time point, the expression of these cancer-related genes could be characterized into four clusters: genes that were upregulated during the early stage and downregulated during the late stage (Cluster 1), genes that upregulated at all times (cluster 2), genes that were kept downregulated at all times (cluster 3), and genes that were stable through all the stages (cluster 4) (Figure 4B and Supplementary Table S4). Furthermore, we validated the gene expression levels of CCND2, CD163, CADM1, L3MBTL4, BCL71, and IGF1 by qRT-PCR, and the results revealed a high degree of consistency with the results from the RNA-Seq analysis (Figures 4B,D–I). In addition, we confirmed that the expression of the CCND2 and CD163 genes was significantly elevated in the OSCC tissue compared with that of normal oral cavity squamous cells, according to the Oncomine database analysis (Figure 4C).

### Dynamic Gene Expression in the GMSCs With or Without *F. nucleatum* Infection

**FIGURE 5:**
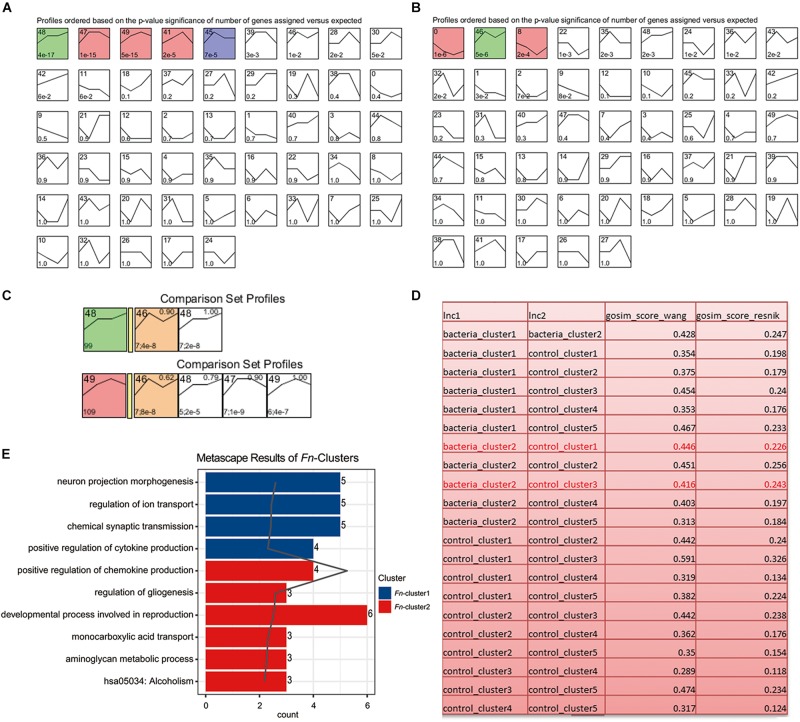
Short Time-series Expression Miner (STEM) analysis of the GMSCs. **(A)** Without *F. nucleatum* infection or **(B)** with *F. nucleatum* infection. The colored boxes represent significantly enriched profiles (*P* value < 0.001). **(C)** Functional similarity analysis of the gene clusters in the control (gene clusters 48 and 49) and *F. nucleatum*-stimulated group (gene cluster 46). **(D)** Functional similarity analysis of all gene clusters in the control and the *F. nucleatum*-stimulated groups according to the Wang and Resnik scores. **(E)** Gene function enrichment analysis of the significantly affected genes in the bacteria-induced clusters 0, 8, and 46.

To analyze the dynamic gene variation at different time points (3, 7, 14, and 21 day) during *F. nucleatum* stimulation, we performed a pattern analysis using STEM software, which has been widely used for in-depth analysis of time-course gene expression data. The results revealed three significant expression patterns in the control group (*P* < 0.001): (1) gradually increasing, remaining stable at 7–14 day and before increasing gradually at later time points (green, cluster 48; including ART5, BMP6, CHI3L2, etc.); (2) increasing at 14 day and then decreasing (red, cluster 47, 49 and 41, such as APOBR, AKR1B15, and CHD5); and (3) increasing at 7 day and then decreasing (blue, cluster 45, such as ARC and HID1) (Figure 5A and Supplementary Table S5). The results also revealed two expression patterns in the *F. nucleatum*-stimulated group (*P* < 0.001): (1) gradually decreasing at 14 day and then increasing (red, cluster 0 and 8; including CPNE4, EFNB3, AKNAD1, C1QTNF4); and (2) gradually increasing from 3 to 7 day, decreasing from 7 to 14 day and then increasing gradually from 14 to 21 day (green, cluster 46, including CHI3L2, ASB5, and CTXND1) (Figure 5B and Supplementary Table S6). The genes in clusters 48 and 49 (in the control group) and genes in cluster 46 (in the *F. nucleatum* stimulated group) had the similar trend variances as time increased, and we therefore performed a function analysis among these clusters. The results indicated that the functions of these genes in clusters 48/49 differed from those in cluster 46 (Figures 5C,D). The results from the GO functional analysis showed that a significant number of the DEGs induced by *F. nucleatum* stimulation in clusters 0 and 46 were enriched mainly in cytokine production, transport, gliogenesis, and metabolic processes (Figure 5E and Supplementary Table S7).

### Screening of the Dynamic DEGs After *F. nucleatum* Stimulation in GMSCs

**FIGURE 6:**
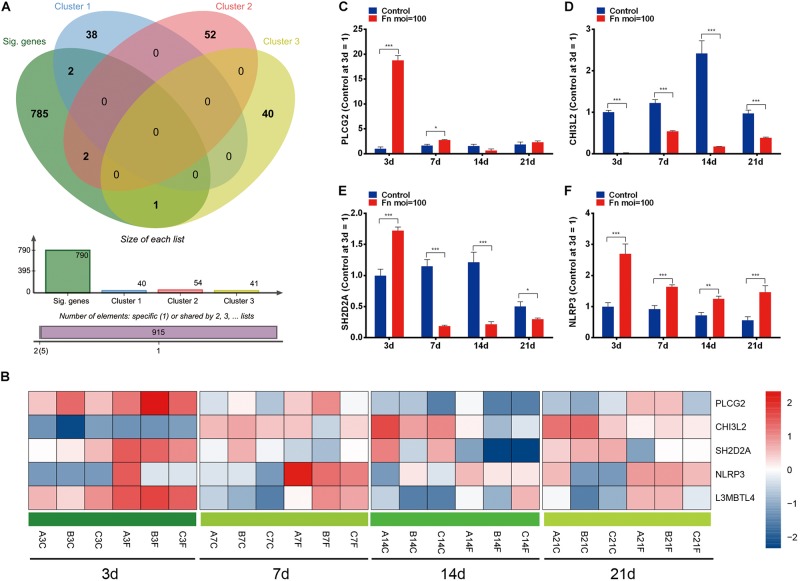
Dynamic DEGs regulated by *F. nucleatum* in a time-dependent manner. **(A)** Venn analysis of time-course DEGs and genes with significantly enriched profiles in *F. nucleatum*-stimulated GMSCs. **(B)** Heatmap of the 5 dynamic DEGs generated by time-series *F. nucleatum* infection. The relative gene expression of the dynamic DEGs: **(C)** PLCG2, **(D)** CHI3L2, **(E)** SH2D2A, **(F)** NLRP3, as determined by qRT-PCR. ^∗^*P* < 0.05, ^∗∗^*P* < 0.01 and ^∗∗∗^*P* < 0.001.

To identify the dynamic DEGs that were specifically induced by *F. nucleatum* stimulation and the gene expression variances due to increased stimulation, we performed an overlap analysis with the time-course DEGs and the genes in clusters 0, 8, and 46, and the results indicated that five dynamic DEGs (PLCG2, CHI3L2, L3MBTL4, SH2D2A, and NLRP3) were significantly varied in a time-dependent manner (Figures 6A,B). The relative expression levels of the PLCG2, CHI3L2, SH2D2A, NLRP3, and L3MBTL4 genes were validated by qRT-PCR, and the results were concordant with data from the RNA-Seq analysis (Figures 6C–F, 4G).

### Screening of the miRNAs and Transcription Factors (TFs) and Constructing the Integrated Network

**FIGURE 7:**
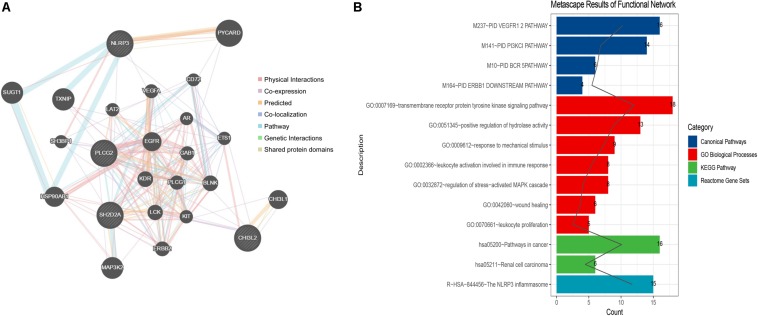
Network construction and gene function analysis based on the five dynamic DEGs. **(A)** The functional network constructed through GeneMANIA. The genes in circles with a white slash are the dynamic DEGs. The dynamic DEGs and the predicated genes interact based on physical interactions, co-expression, predicated, co-localization, pathway, genetic interactions, and shared protein domains. **(B)** The functional enrichment analysis of all genes in the network based on the Metascape database.

**FIGURE 8:**
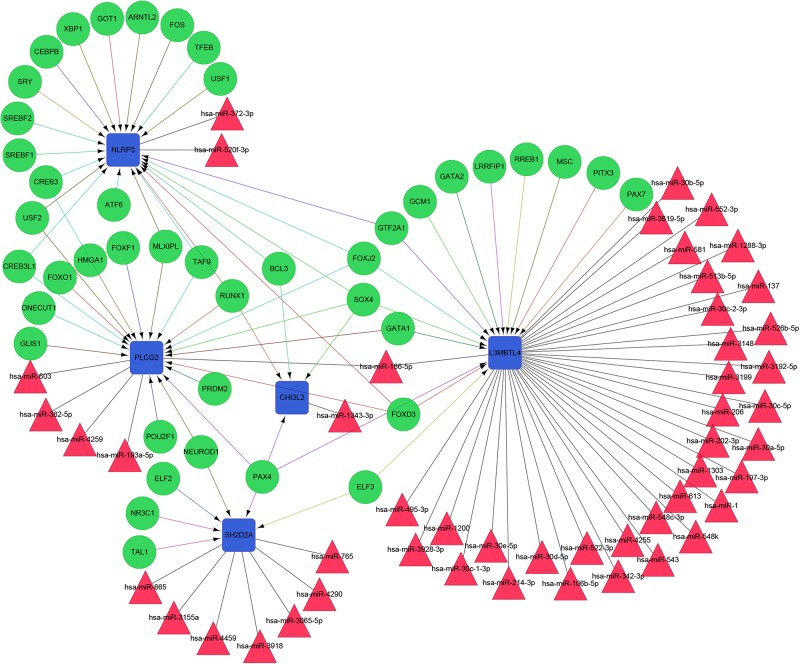
The TF-miRNA-mRNA Integrated network based on the five dynamic DEGs. Green nodes represent transcription factors; red triangles indicate miRNAs; blue boxes indicate mRNA genes; arrows represent transcriptional regulatory relationships; and lines represent miRNA regulatory relationships.

The constructed functional network based on the gene function predictions of the five dynamic DEGs, according to the GO (Biological Process) annotation patterns as determined through GeneMANIA, is shown in Figure 7A. The results indicated that 24 genes, including VEGFA, CD72, MAP3K2, and PLCG1, were enriched in this network based on their functions related to physical interactions, co-expression, co-localization, pathway, genetic interaction, and shared protein domains. In addition, the results from the functional enrichment analysis demonstrated that among the 24 genes predicated, 12 genes were enriched in cancer-related pathways (Figure 7B and Supplementary Table S8). According to the miRWalk 2.0 database, miRNAs targeting dynamic DEGs [such as miRNA (miR)-372-3p→NLRP3, miR-382-5p→PLCG2, miR-665→SH2D2A, miR-30c-2-3p→L3MBTL4, and miR-1343-3p→CHI3L2] were enriched. With the iRegulon plug-in, the transcriptional regulatory relationships among these five DEGs were searched and identified. In the transcriptional regulatory network, NLRP3 was targeted by 21 TFs, such as CREB3, SREBF1, and SRY; PLCG2 was targeted by 18 TFs, including FOXF1, TAT9, RUNX1; CHISL2 was targeted by four TFs, such as RUNX1, BCL3, SOX4 and PAX4; L3MBTL4 was targeted by 13 TFs, such as SOX4, RREB1 and MSC; and SH2D2A was targeted by six TFs, including TAL1, NR3C1, ELF2, NEOROD1, PAX4 and ELF3. Finally, the TF-miRNA-mRNA regulatory networks were constructed (Figure 8 and Supplementary Table S9).

## Discussion

The role of *F. nucleatum* on tumorigenesis has been widely recognized, but the long-term effects of *F. nucleatum* on normal cells had not been revealed in full. In this study, we identified 790 time-course DEGs that were influenced by *F. nucleatum* in a time-dependent manner during the osteogenic differentiation process of GMSCs. Interestingly, we found that the time-course DEGs were significantly enriched in cancer-related signaling pathways, and 50 TAGs were identified. In addition, PLCG2, CHI3L2, L3MBTL4, SH2D2A, and NLRP3 were specifically recognized as dynamic DEGs with increasing *F. nucleatum* stimulation time. Based on these 5 genes, the integrated function network was constructed, which included 24 proteins and 12 genes in cancer-related pathways. Furthermore, we found that 43 TFs and 50 miRNAs were targeted to these five DEGs. Our study suggests that *F. nucleatum* might play roles in promoting tumorigenesis during the long-term stimulation of GMSCs.

A previous study showed that *F. nucleatum* is closely related to the occurrence of cancers and can enhance the malignant tumor induced environment (Amitay et al., 2017). *F. nucleatum* is prominent in human colorectal cancers and distal metastases lesions (Bullman et al., 2017). *F. nucleatum* promotes colorectal cancer cell proliferation and tumorigenesis by activating Toll-like receptor 4(TLR4) signaling, which upregulates nuclear factor-κB, as indicated by increased MicroRNA-21 expression or the TLR4/p-PAK1/p-β-catenin S675-cascade signaling pathway (Yang et al., 2017; Wu et al., 2018). *F. nucleatum* promotes chemoresistance by upregulating BIRC3 expression and modulating autophagy in colorectal cancer (Yu et al., 2017; Zhang et al., 2019). In addition, in esophageal cancer tissue, *F. nucleatum* was associated with shorter times of survival for patients, which suggested the *F. nucleatum* has the potential to become as a prognostic biomarker (Yamamura et al., 2016). *F. nucleatum* levels were also significantly enriched in patients with OSCC (Pushalkar et al., 2012). *F. nucleatum* can induce the production of various matrix metalloproteinases (MMPs), such as MMP-9 and MMP-13, which have been used in monitoring and detecting metastatic phenotypes in oral cancer (Patel et al., 2007; Kao et al., 2009).

In gingival epithelial cells (GECs), *F. nucleatum* could activate the NLRP3 inflammasome through the activation of caspase-1 and elevate the damage-associated molecular patterns, including high-mobility group box-1 protein (HMGB1) and apoptosis-associated speck-like protein, which induce NF-κB signaling pathway activation and the secretion of mature IL-1β (Bui et al., 2016). NLRX1, a member of the nod-like receptor family that localizes to mitochondria, could positively regulate ATP-mediated NLRP3 inflammasome activation through ROS in the GECs, and has the potential to participate in anti-microbial responses of cells in either a healthy or diseased states in the oral cavity (Hung et al., 2018). NLRP3 plays key roles in the pathogenesis of periapical periodontitis, and *F. nucleatum* is one of the crucial microorganisms that might activate the inflammasome in periapical tissues (Ran et al., 2017). In the future, we will perform more studies to explore the effect of *F. nucleatum* on GMSCs to clarify the defined roles of *F. nucleatum* in oral infection diseases.

Phospholipase C gamma 2 (PLCG2) plays key roles in B-cell survival and proliferation, and a mutation in the PLCG2 gene can protect mice from *Helicobacter*-induced gastric mucosa-associated lymphoid tissue lymphoma (Gossmann et al., 2016). Human cartilage chitinase 3-like protein 2 (CHI3L2, also known as YKL-39) is a member of the glycosyl hydrolases families that lacks chitinase activity. In glial tumors, CHI3L2 was increased and then enhanced the phosphorylation level of ERK1/2, which finally resulted in the inhibition of cell mitogenesis and proliferation (Areshkov et al., 2012). L3MBTL4 is a potential tumor suppressor gene in chromosome arm 18p. The gene is prone to mutations by deletion or breakage, and its expression level is downregulated in breast tumors (Addou-Klouche et al., 2010). SH2D2A plays an important modulatory role in T-cell mediated immune surveillance of cancer and may lead to defective control and elimination of autoreactive T cells (Uncini et al., 2011; Berge et al., 2012). In the current study, we confirmed that *F. nucleatum* can significantly upregulate the gene expression of NLRP3, PLCG2, CHI3L2, L3MBTL4, and SH2D2A in GMSCs, which suggests that *F. nucleatum* infection may induce GMSC differentiation that deviates from that in normal cells. Our study is limited by the lack of biological experiments and clinical data to validate the effects of *F. nucleatum* on human tumor growth, and any possible tumorigenic activity will be observed *in vivo* in future studies.

## Conclusion

In conclusion, our study confirms that *F. nucleatum* can regulate the gene expression of TAGs in GMSCs during long-term stimulation. The gene expression levels of PLCG2, CHI3L2, L3MBTL4, SH2D2A, and NLRP3 were time related, associated with *F. nucleatum-*infected GMSCs, and were accompanied by several miRNAs (such as miR-372-3p, miR-603, and miR-495-3p) and TFs, including CREB3, GATA2, and SOX4.

## Data Availability Statement

The datasets generated for this study can be found in the GSE126821. Other data used to support the findings of this study are included in the Supplementary Material. If any other data are needed, please contact the corresponding author.

## Ethics Statement

The studies involving human participants were reviewed and approved by the Medical Ethical Committee of School of Stomatology, Shandong University. The patients/participants provided their written informed consent to participate in this study.

## Author Contributions

QF conceived, designed, and supervised the study. WK, DT, and JZ collected the samples. WK and TS performed the experiments, analyzed the data, and wrote the manuscript. WK, TS, and QF revised the manuscript.

## Conflict of Interest

The authors declare that the research was conducted in the absence of any commercial or financial relationships that could be construed as a potential conflict of interest.

## Footnotes

1 https://www.ncbi.nlm.nih.gov/geo/

2 http://ctdbase.org/

3 http://jvenn.toulouse.inra.fr/app/example.html

4 https://www.oncomine.org/resource/main.html

5 http://metascape.org/gp/index.html#/main/step1

6 http://genemania.org/search

7 http://zmf.umm.uni-heidelberg.de/apps/zmf/mirwalk2/

8 http://apps.cytoscape.org/apps/iRegulon

## Supplementary Material

FIGURE S1 Data visualization according to cluster analysis. The gene expression profiles of the 24 samples are displayed. All gene expression levels in each sample are shown with different colored lines.

FIGURE S2 Pathview analysis of the axon guidance signaling pathway. The genes in the red rectangle are regulated by time-series *F. nucleatum* stimulation. The genes encircled in green are carcinogenesis-related DEGs.

FIGURE S3 Pathview analysis of the adherens junction signaling pathway.

FIGURE S4 Pathview analysis of the epithelial cell signaling in helicobacter pylori infection signaling pathway.

FIGURE S5 Pathview analysis of the viral carcinogenesis signaling pathway.

FIGURE S6 Pathview analysis of the renal cell carcinoma signaling pathway.

TABLE S1 Primer sequences for quantitative real-time PCR of the GMSCs.

TABLE S2 The gene symbols of all 790 DEGs.

TABLE S3 GO enrichment analysis of all co-regulated DEGs.

TABLE S4 Fifty overlapped tumor-associated genes (TAGs) between time-series DEGs and all the TAGs in the CTD database.

TABLE S5 Five significant gene expression clusters in the control group according to STEM analysis (*P* < 0.001).

TABLE S6 The genes in three significant gene expression clusters in the *F. nucleatum*-treated group according to STEM analysis (*P* < 0.001).

TABLE S7 The annotation and function enrichment analysis results for the genes in three significant clusters in the *F. nucleatum*-treated group according to STEM analysis.

TABLE S8 The annotation and function enrichment analysis results for the genes in the function network constructed by GeneMANIA.

TABLE S9 All the miRNAs and TFs targeting to the five dynamic DEGs (PLCG2, CHI3L2, L3MBTL4, SH2D2A, and NLRP3).
