# Supplementary figures and images for: Time-Course Transcriptome Analysis of Gingiva-Derived Mesenchymal Stem Cells Reveals That Fusobacterium nucleatum Triggers Oncogene Expression in the Process of Cell Differentiation
Source: Front Cell Dev Biol. 2020 Jan 14;7:359. doi: 10.3389/fcell.2019.00359 (PMC6970952; doi:10.3389/fcell.2019.00359)

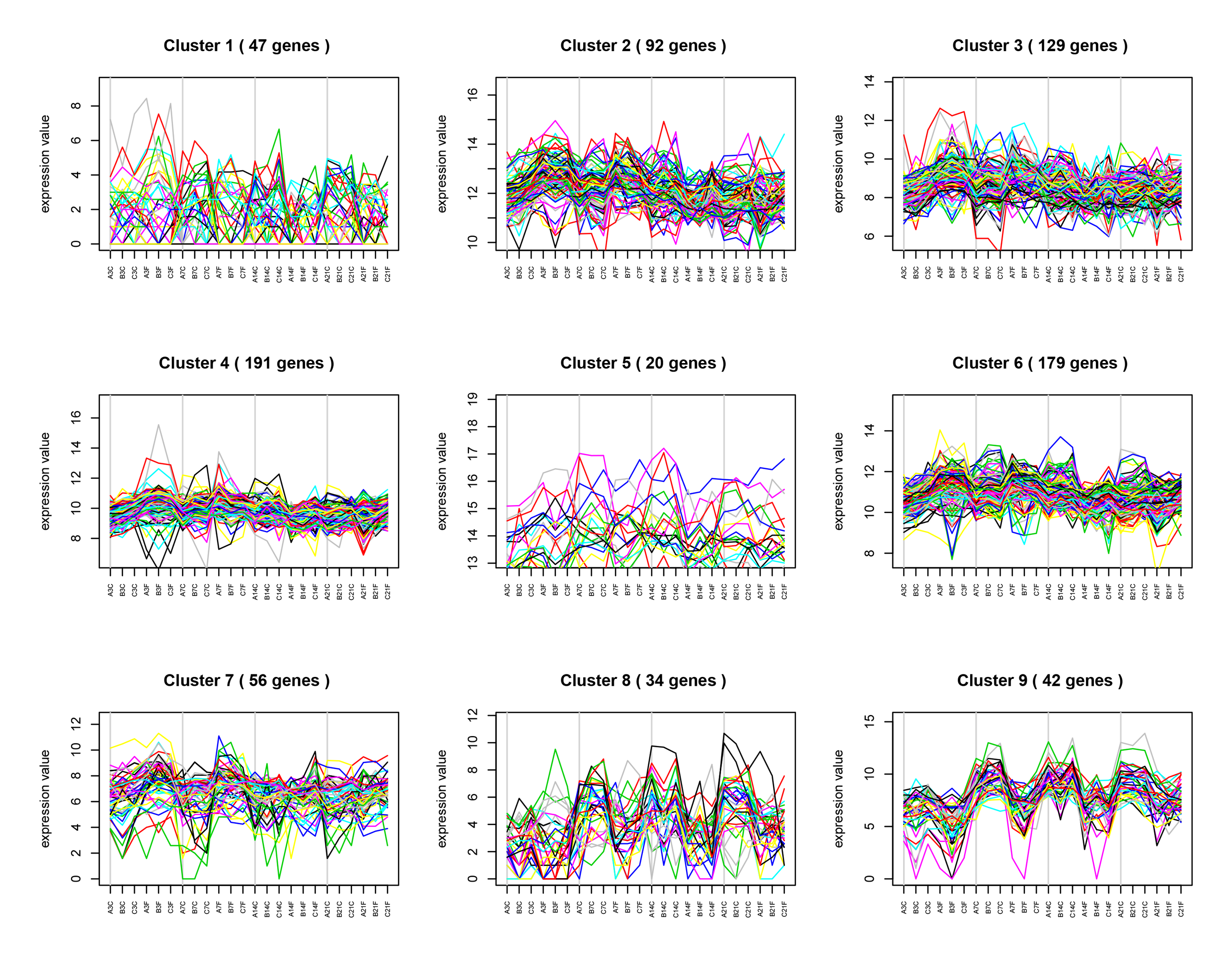

Supplement: FIGURE S1 — Data visualization according to cluster analysis. The gene expression profiles of the 24 samples are displayed. All gene expression levels in each sample are shown with different colored lines. [file Image_1.TIF]

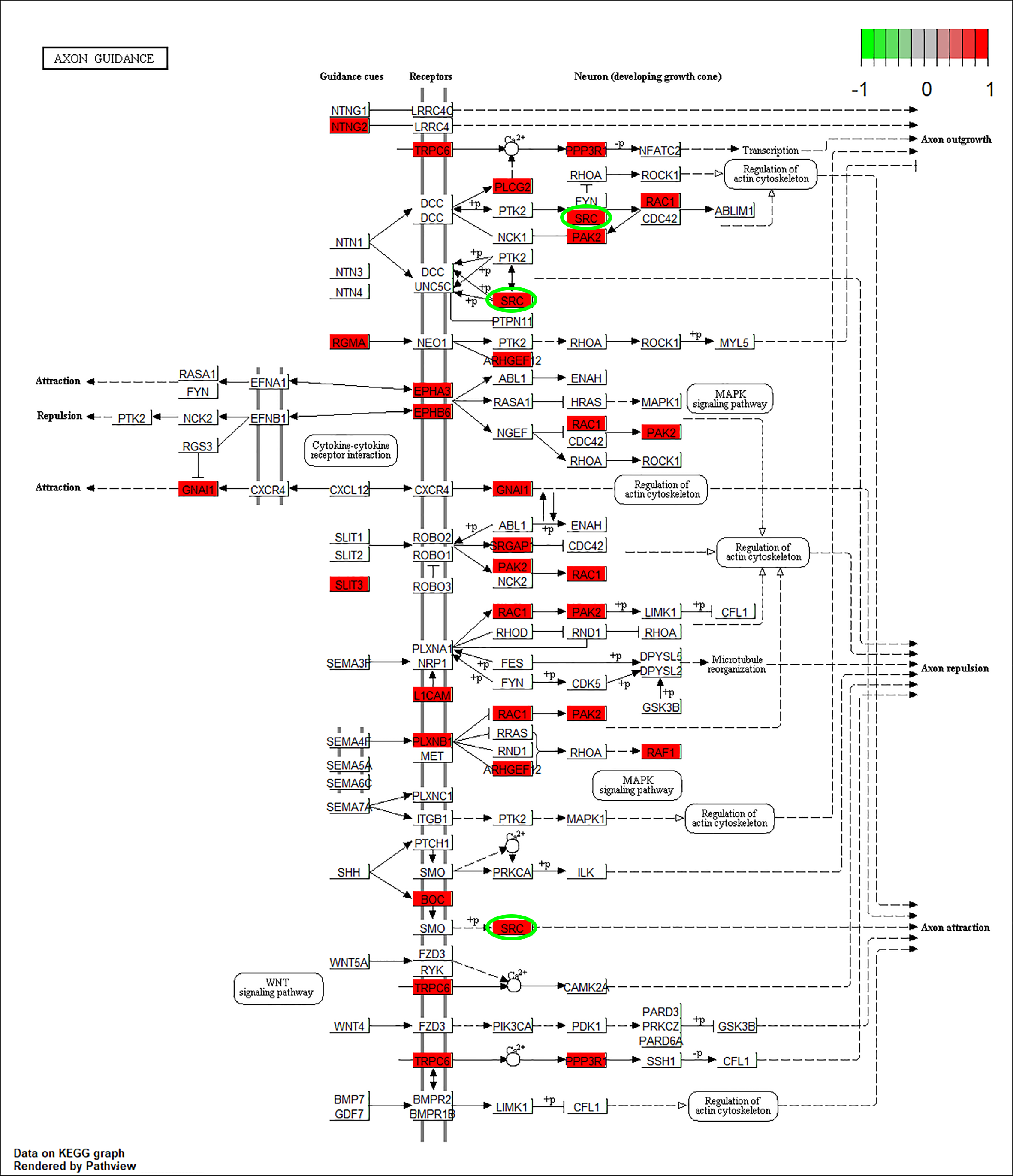

Supplement: FIGURE S2 — Pathview analysis of the axon guidance signaling pathway. The genes in the red rectangle are regulated by time-series F. nucleatum stimulation. The genes encircled in green are carcinogenesis-related DEGs. [file Image_2.TIF]

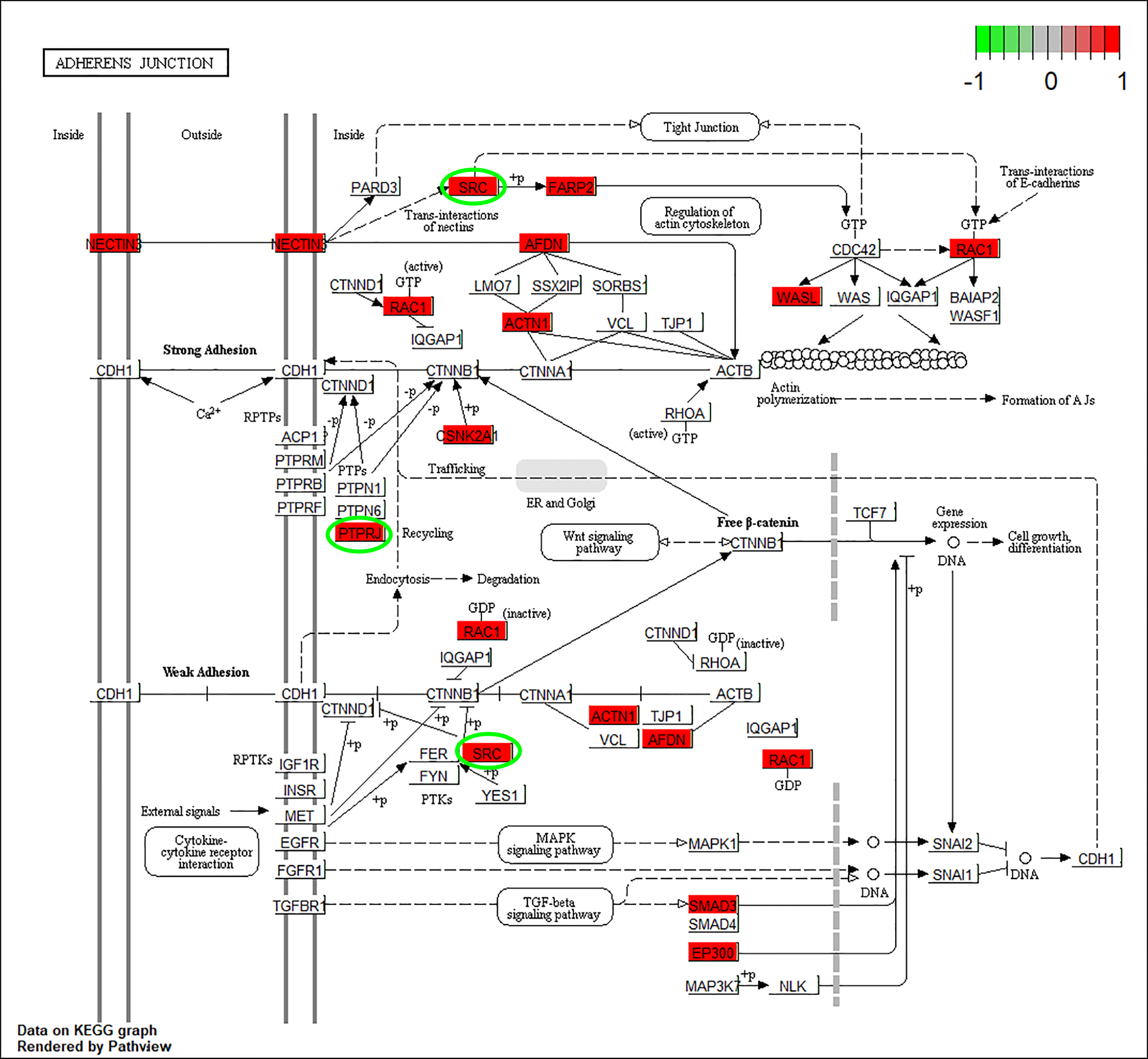

Supplement: FIGURE S3 — Pathview analysis of the adherens junction signaling pathway. [file Image_3.TIF]

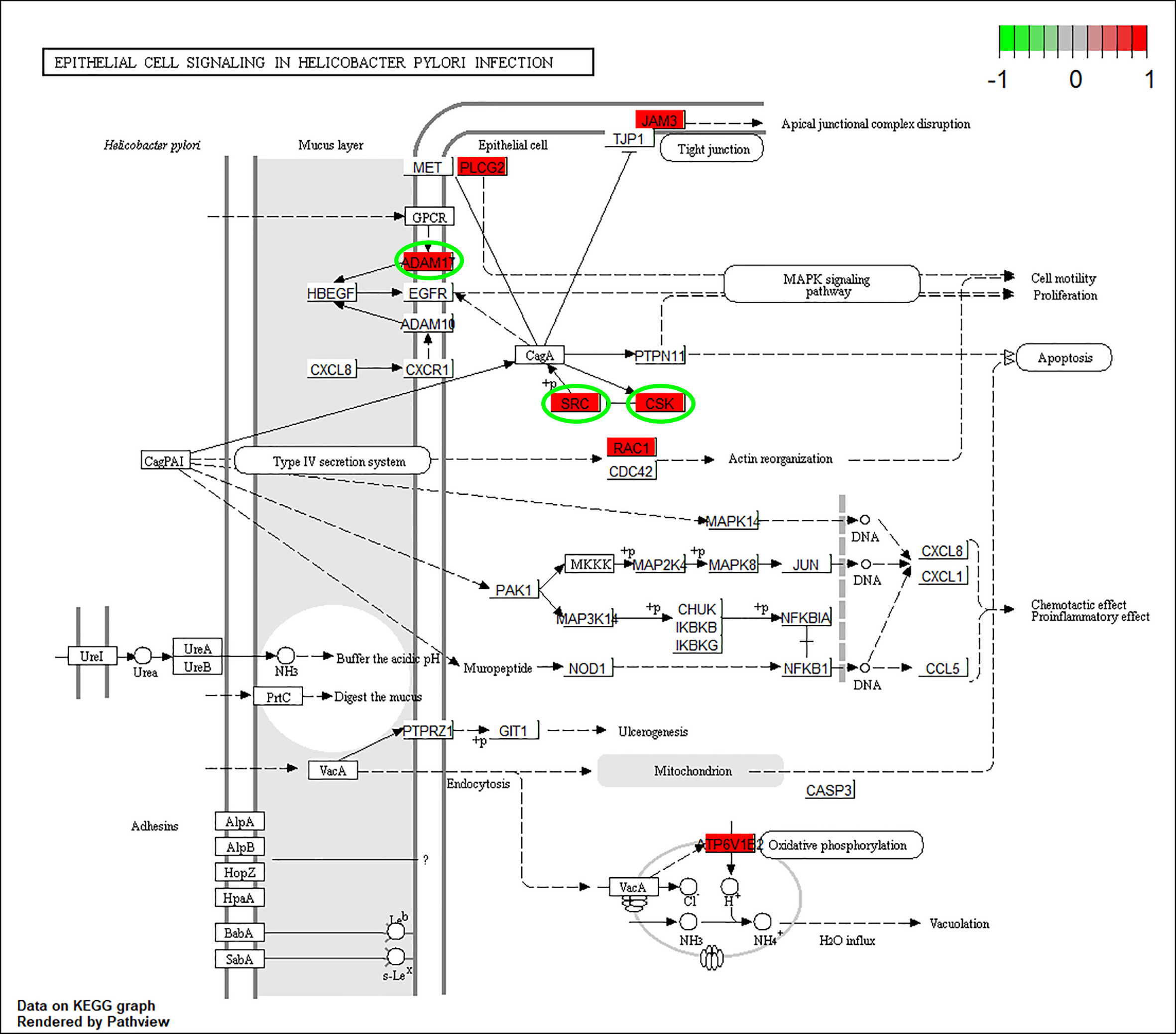

Supplement: FIGURE S4 — Pathview analysis of the epithelial cell signaling in helicobacter pylori infection signaling pathway. [file Image_4.TIF]

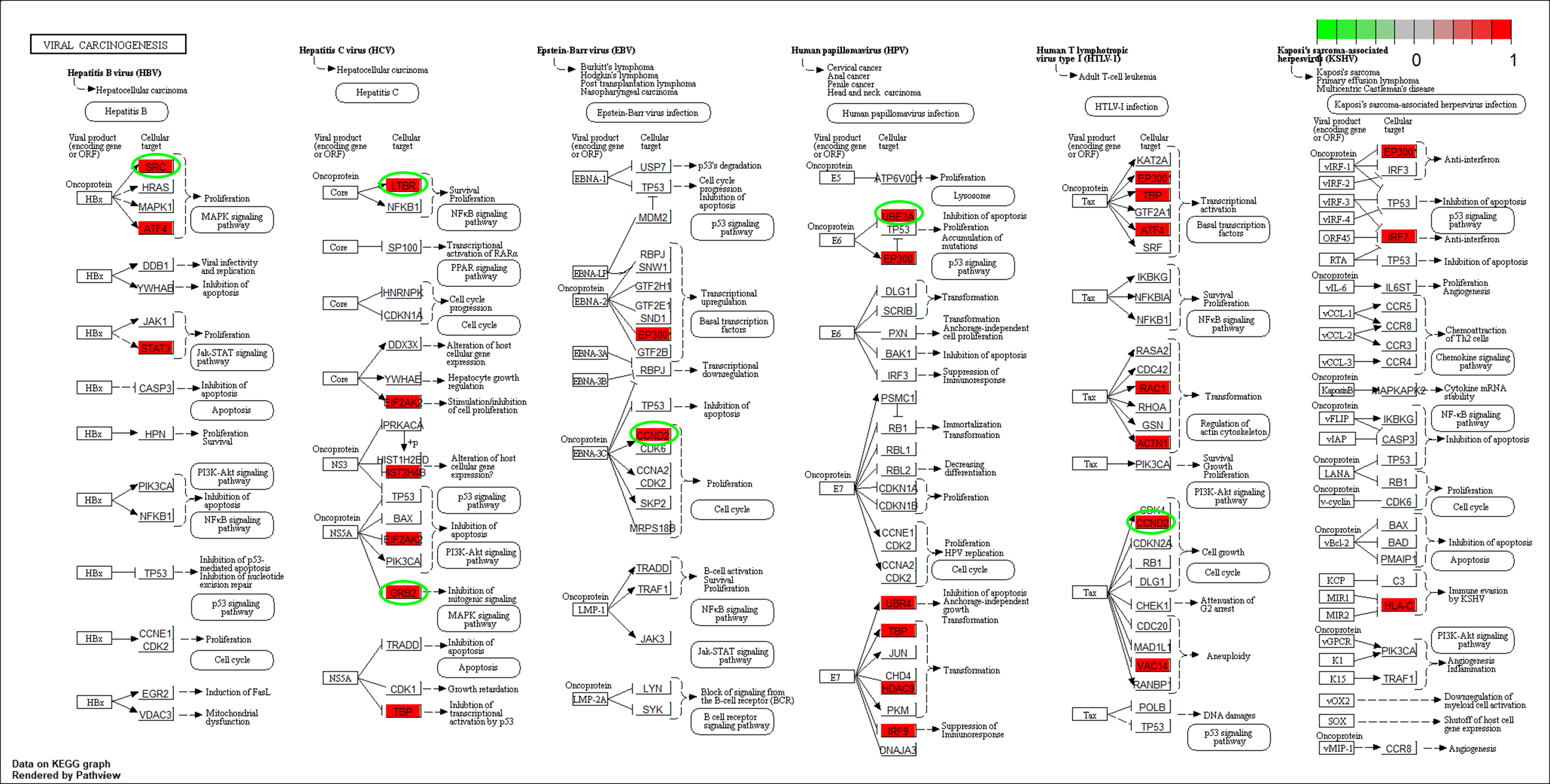

Supplement: FIGURE S5 — Pathview analysis of the viral carcinogenesis signaling pathway. [file Image_5.TIF]

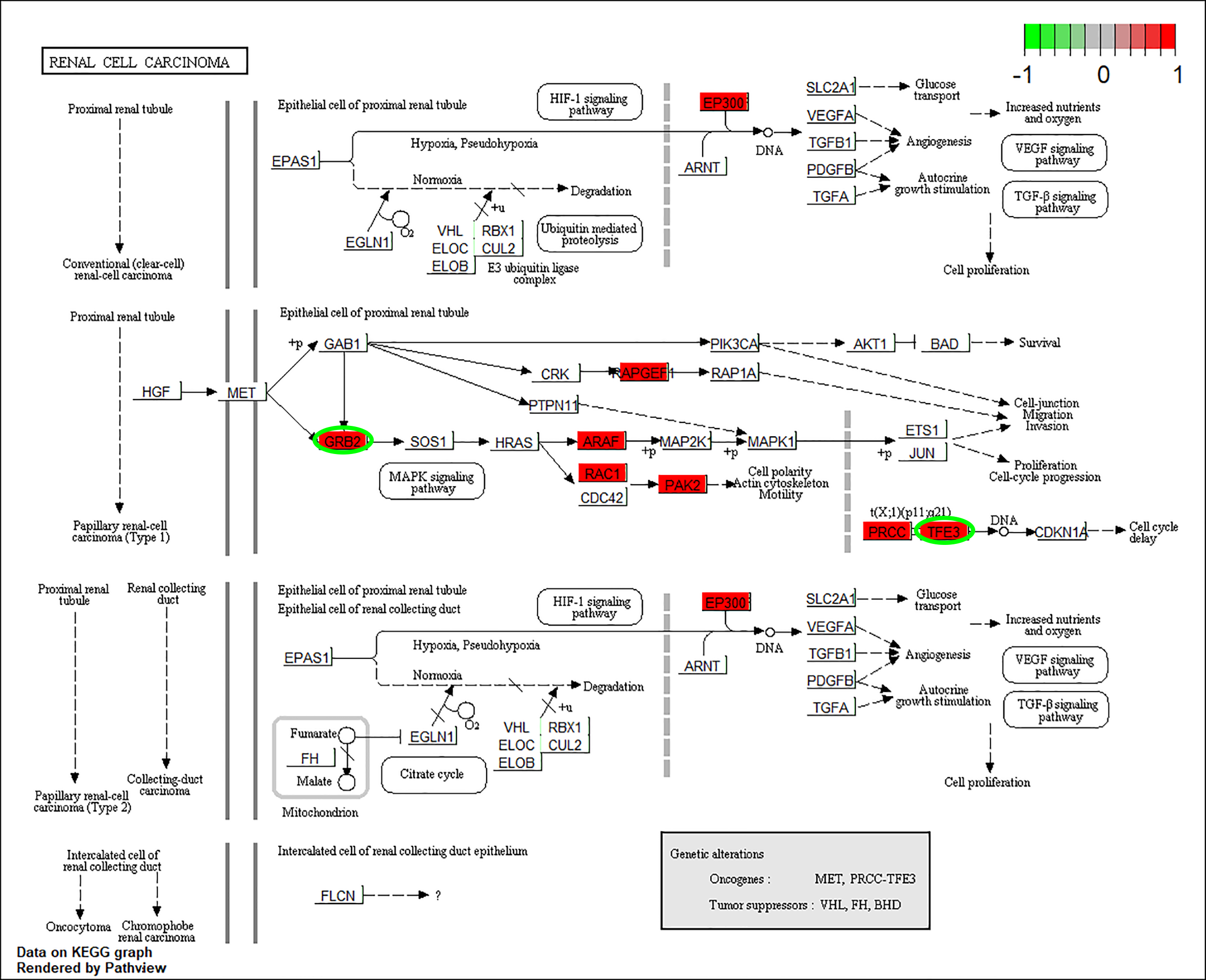

Supplement: FIGURE S6 — Pathview analysis of the renal cell carcinoma signaling pathway. [file Image_6.TIF]
