# Supplementary material for: Time-Course Transcriptome Analysis of Gingiva-Derived Mesenchymal Stem Cells Reveals That Fusobacterium nucleatum Triggers Oncogene Expression in the Process of Cell Differentiation
Source: Front Cell Dev Biol. 2020 Jan 14;7:359. doi: 10.3389/fcell.2019.00359 (PMC6970952; doi:10.3389/fcell.2019.00359)
Supplement: TABLE S1 — Primer sequences for quantitative real-time PCR of the GMSCs. [file Table_1.DOCX]

**Table S1. Primers sequences for quantitative real-time PCR (qRT-PCR)**

| Gene | Primer sequences | | |
| --- | --- | --- | --- |
|  | 5ˊ~3ˊForward | 5ˊ~3ˊReverse | |
| GAPDH  CCND2  CD163  CADM1  L3MBTL4  BCL7  IGF1  PLCG2  CHI3L2  SH2D2A  NLRP3 | GCACCGTCAAGGCTGAGAAC  CACCTGGATGCTGGAGGTCTG  TCTCTTGGAGGAACAGACAAGG  GAGCTAAAAGGCAAATCGGAGG  ACCAATGCAGTTCCTGCCAA  AGGCAAGGACGAGAAGTGTG  ATCGTGGATGAGTGCTGCTT  CGAGGCGATGTGGATGTCAA  TGGAGACCAAGGTTCAGTTCTT  TCAGGGGTGCTACTTGGTGC  TGCGATCAACAGGAGAGACC | | TGGTGAAGACGCCAGTGGA  TCAGCGGGCTGGTCTCTTTG  CCTGCACTGGAATTAGCCCA  CTAGATACCGCTGGGTCTGC  CCCAACCATCAAAATGAACCTTTAC  CTGGTTGCTGTTATCGTCATGC  GACAGAGCGAGCTGACTTGG AGTGCCGAGTCCATTTCTGG  CTGGTTGCAGGATTTGCCAG  AGTCCGGCTCCTGTAAGTCAG  CGTGCATTATCTGAACCCCAC |
